# A time series driven model for early sepsis prediction based on transformer module

**DOI:** 10.1186/s12874-023-02138-6

**Published:** 2024-01-25

**Authors:** Yan Tang, Yu Zhang, Jiaxi Li

**Affiliations:** 1 Department of Clinical Laboratory Medicine, Jinniu Maternity and Child Health Hospital of Chengdu, Chengdu, China; 2 Information Center, West China Hospital, Sichuan University, Chengdu, China

**Keywords:** Sepsis, Transformer, Time-series, SHAP, Predicting, Sepsis

## Abstract

Sepsis remains a critical concern in intensive care units due to its high mortality rate. Early identification and intervention are paramount to improving patient outcomes. In this study, we have proposed predictive models for early sepsis prediction based on time-series data, utilizing both CNN-Transformer and LSTM-Transformer architectures. By collecting time-series data from patients at 4, 8, and 12 h prior to sepsis diagnosis and subjecting it to various network models for analysis and comparison. In contrast to traditional recurrent neural networks, our model exhibited a substantial improvement of approximately 20%. On average, our model demonstrated an accuracy of 0.964 (± 0.018), a precision of 0.956 (± 0.012), a recall of 0.967 (± 0.012), and an F1 score of 0.959 (± 0.014). Furthermore, by adjusting the time window, it was observed that the Transformer-based model demonstrated exceptional predictive capabilities, particularly within the earlier time window (i.e., 12 h before onset), thus holding significant promise for early clinical diagnosis and intervention. Besides, we employed the SHAP algorithm to visualize the weight distribution of different features, enhancing the interpretability of our model and facilitating early clinical diagnosis and intervention.

## Introduction

Sepsis is defined as life-threatening organ dysfunction caused by a dysregulated host response to infection [1]. Failure to promptly recognize and intervene with treatment can result in multiple organ dysfunction or even fatality. Over the last few decades, due to the timely administration of antibiotics, fluid resuscitation, and multi-organ support therapies, the mortality rate associated with sepsis has progressively diminished. Nevertheless, the mortality rate still persists at a high level. The Global Burden of Disease report for the year 2017 revealed a global total of 48.9 million reported cases of sepsis, with a mortality rate of 22.5%, accounting for nearly 20% of the total global deaths [2–4]. The progression speed of sepsis can vary due to individual differences and the type of infection, but typically, certain signs and symptoms might emerge during the initial stages of its onset. Acknowledging the non-absolute nature of these indicators is paramount, given that diverse patients may present varying signs. Moreover, the early symptoms of sepsis may sometimes overlap with symptoms of other diseases. Therefore, early prediction of sepsis is crucial for a comprehensive assessment and diagnosis of the condition's progression.

As sepsis patients advance through various disease stages, the precise early prediction of a patient's potential progression to sepsis during the initial phases of inflammation is critically significant for clinical practitioners. This significance arises from its dual role in not only facilitating the evaluation of disease severity but also in enhancing treatment strategies, mitigating unfavorable outcomes, and ultimately prolonging patients' lives. Currently, various clinical scoring systems, such as the Sequential Organ Failure Assessment (SOFA) score [5], the Acute Physiology and Chronic Health Evaluation (APACHE-II) score [6], and the Predisposition, Insult/Infection, Response, and Organ Dysfunction (PIRO) score [7], can assist clinical practitioners in evaluating patients' overall risk and prognosis. Nonetheless, these scoring systems are designed for a broader range of critically ill patients and are not specifically tailored to sepsis. To prevent the progression of severely infected patients into sepsis, pre-diagnosis and continuous monitoring of sepsis are of utmost importance, potentially leading to improved patient survival rates.

In recent times, machine learning has played a pivotal role in medical research, facilitating the creation of predictive models customized to meet specific clinical requirements and data attributes. In contrast to traditional clinical scoring systems, machine learning models possess the ability to consistently learn from feedback, enabling a gradual enhancement of their performance over time—an achievement that traditional scoring systems might struggle to emulate. The construction of existing sepsis prediction models heavily relies on the utilization of machine learning models. For instance, Calvert et al. [8] introduced the InSight algorithm, which utilizes 9 parameters—8 routine vital signs and patient age—to predict sepsis which achieved an Area Under the Receiver Operating Characteristic curve (AUROC) of 0.72. Yao et al. [9] found that the xgboost model outperforms traditional logistic regression models and SOFA scores in both discrimination and calibration in early identification of high-risk sepsis mortality patients, with AUCs of 0.835, 0.737, and 0.621 respectively. Beyond conventional machine learning algorithms, there have been endeavors within research to explore enhancements utilizing deep learning algorithms. For a prediction 3 h prior to sepsis onset, Matthieu Scherpf et al. [10] exploited a recurrent neural network and achieved an AUROC of 0.81.

Nonetheless, most studies face limitations as they tend to narrow their focus on only a handful of critical patient characteristics or depend on data from specific timeframes or intervals, unintentionally neglecting the dynamic evolution of potential clinical features and disease conditions. The signs and symptoms observed before the onset of sepsis are critically important in the development of sepsis. Hence, we propose a Transformer-based time-series data prediction model that collects clinical features of patients before illness onset, assesses the influence of various retrospective timeframes, and enhances interpretability with the Shap algorithm [11]. This approach aims to investigate clinical factors related to sepsis onset, offering clinicians guidance for effective intervention measures within the early sepsis onset window.

## Data collection and preprocessing

### Data collection

The eICU Collaborative Research Database is a large, multi-center intensive care unit (ICU) database (https://eicu-crd.mit.edu/about/eicu/) established through a collaboration between the Massachusetts Institute of Technology (MIT) and Philips Group [12]. The database contains clinical data of over 200,000 patients from 208 hospitals in the United States, recorded between 2014 and 2015. It includes demographic information, vital signs, laboratory test results, treatments, diagnoses, and other data. The data quality is high, validated through multiple research studies. This research does not involve any ethical concerns and consent to participate.

**Fig. 1:**
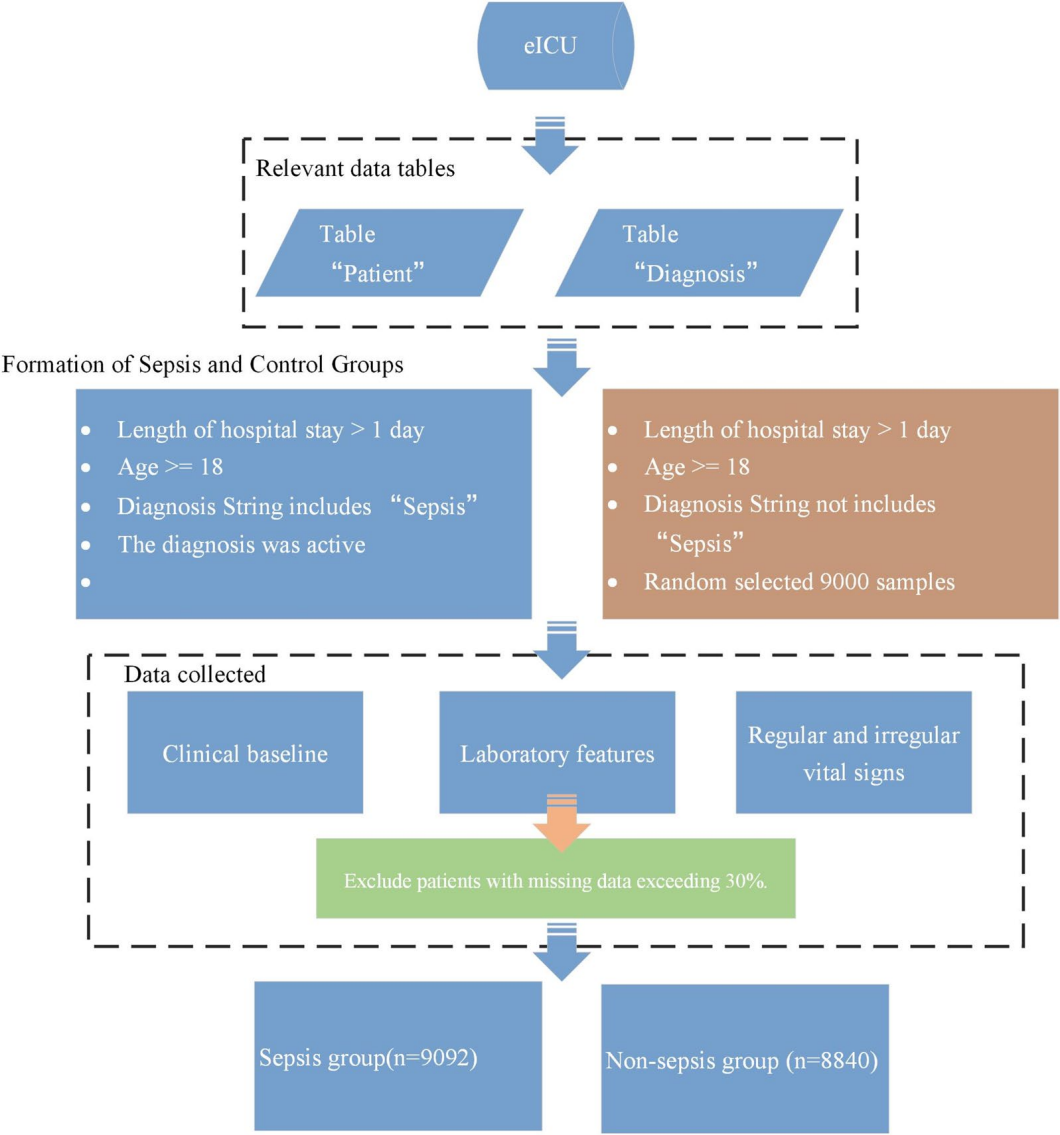
Data inclusion and exclusion process

Based on the patient table and diagnosis table extracted from the eICU database, we conducted essential patient inclusion and exclusion operations, selecting patients whose diagnosis includes the keyword "sepsis," and ensuring their diagnostic status is "valid" upon discharge. We also ensured that patients were 18 years of age or older. Furthermore, given that we are establishing a time-series model, it was necessary to collect data for patients over a specific period. Therefore, we opted for patients with a hospital stay exceeding one day. We gathered clinical baseline information, all test data during their hospitalization, and vital sign data (both regular and irregular), which served as the foundation for the time-series model. The distinction between the control group and the sepsis group lies in the absence of any "sepsis" incidents during the hospitalization of patients in the control group. Therefore, we randomly selected 9,000 samples as the control group and excluded patients with missing data exceeding 30% based on the results. In the end, the sepsis group included 9,092 individuals, while the control group consisted of 8,840 individuals (as illustrated in Fig. 1). Additionally, we have collected 38,895 external test cases from the MIMIC database following the same standards (Non-sepsis: 34,345, Sepsis: 4550) to validate the effectiveness of the model.

Using the Python Scikit-learn toolkit (scikit-learn: machine learning in Python — scikit-learn 1.3.0 documentation) [13], we split the entire training dataset into a 70:30 ratio for the training and testing sets. Additionally, we conducted multiple cross-validation tests using k-fold (k = 5) [14] to obtain the average performance results of the model.

### Data preprocessing

Due to our study is focused on predicting the occurrence of sepsis in advance based on time-series data, we use the diagnosis time of sepsis patients as a reference point and collect patient time-series data in forward increments of 12 h, 8 h, and 4 h to establish predictive models. The eICU database records data in minutes relative to the time of admission, creating a timeline of patient data. During the process of handling time-series data, we need to establish the aligned time intervals. The start time is uniformly set as the timestamp of the patient's first recorded data, while the end time is adjusted based on the different prediction time windows. Within the specified time range, we have established a uniformly spaced time axis to ensure data alignment at identical time points.

**Fig. 2:**
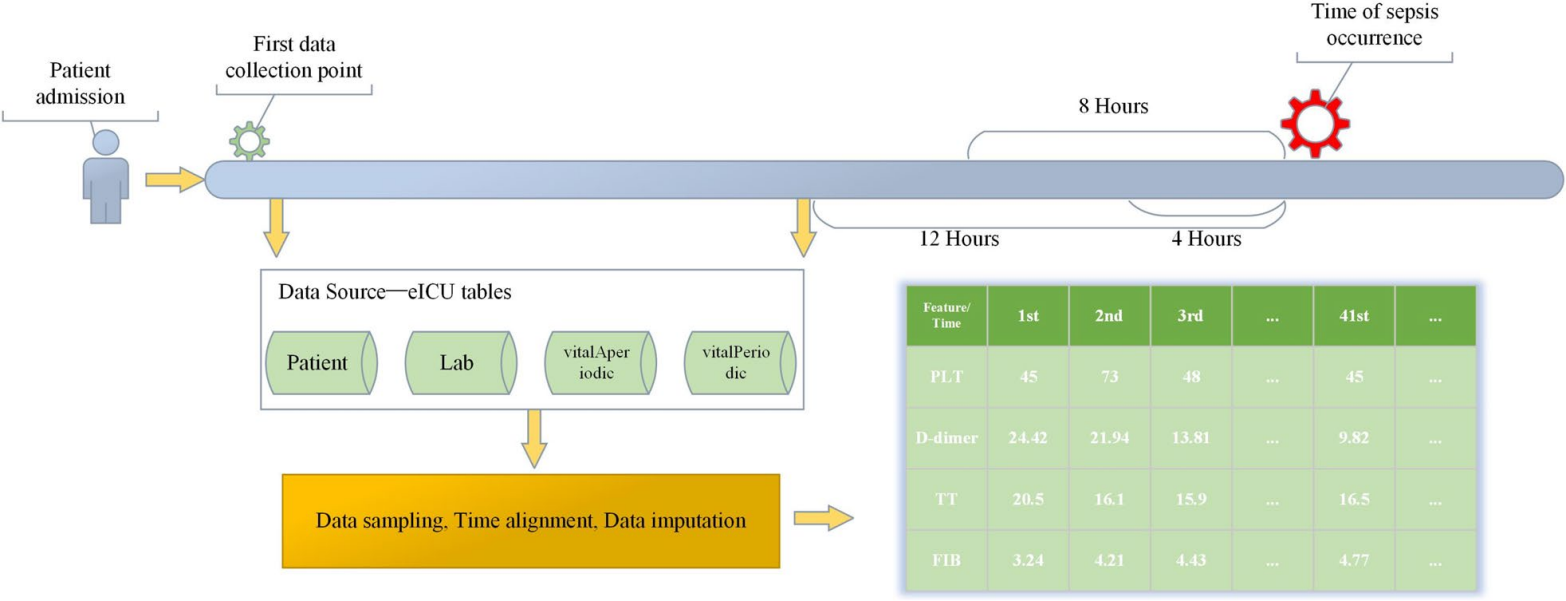
Data processing workflow

To control the scale of input data, we implemented the following procedures: setting the data sampling points to be one hour apart to ensure data alignment at the same time points; collecting data from various sources, and identifying the closest time point on the time axis and aligned the data to that specific time point based on the timestamps associated with each data point; using a forward-fill approach to supplement missing data that might occur during the alignment process when data for a particular time point was absent to maintain data continuity and applying mean imputation to ensure data completeness for patients with entirely missing features. The specific process is as follows (Fig. 2):

Utilizing the Python Scikit-learn toolkit (scikit-learn: machine learning in Python — scikit-learn 1.3.0 documentation) [13], we partitioned the preprocessed data into training and testing sets, maintaining a 7:3 ratio. This ensured that 70% of the data was allocated to the training set, with the remaining 30% dedicated to the testing set. To facilitate unbiased model selection and hyperparameter optimization, we adopted a fivefold cross-validation approach [14]. It is crucial to note that the test set, employed for the final model evaluation, remained entirely independent and played no role in the model selection or parameter optimization processes.

## Model construction

With the widespread adoption of electronic medical record systems, healthcare institutions have accumulated a vast amount of time-series data, which contains rich clinical information. Conventional predictive modeling approaches are confronted with the formidable task of effectively modeling extensive and intricate time-series data. To harness these data more effectively, deep learning models have emerged, capable of automatically learning complex patterns and features within the data. Over the past few years, RNN (Recurrent Neural Network) and their variants, such as LSTM (Long Short-Term Memory), as well as Transformer models, have been widely applied to clinical prediction tasks. By considering the temporal dependencies between observed outcomes, they effectively model time-series data.

RNN: Recurrent Neural Networks are a type of neural network model based on a cyclic structure [15], designed for processing sequential data. Their core concept involves the transmission and retention of information across time dimensions through recurrent connections. An RNN consists of recurrent units, with each unit receiving input from the current time step as well as the hidden state from the previous time step, and it outputs the hidden state for the current time step. This architecture enables RNNs to leverage context information and capture temporal dependencies within sequences.

LSTM: Long Short-Term Memory networks are an improved variant of recurrent neural network architecture designed to address the issues of gradient vanishing and exploding gradients in traditional RNNs [16]. LSTM introduces gate mechanisms, including the input gate, forget gate, and output gate. The input gate determines how much new information should be added to the hidden state of the current time step, the forget gate controls how much of the previous time step's hidden state should be forgotten, and the output gate regulates how much information should be output from the hidden state of the current time step. Through these gate mechanisms, LSTM can effectively capture long-term dependencies while mitigating the problems associated with gradient vanishing and exploding gradients.

With the introduction of Transformer models [17], deep learning has witnessed a significant breakthrough in the field of time-series data modeling. Transformer models, through their self-attention mechanism, can capture global dependencies, mitigating the issues of information loss and gradient vanishing encountered in traditional RNN models. Consequently, they exhibit advantages in handling long-term dependencies and large-scale time-series data.

**Fig. 3:**
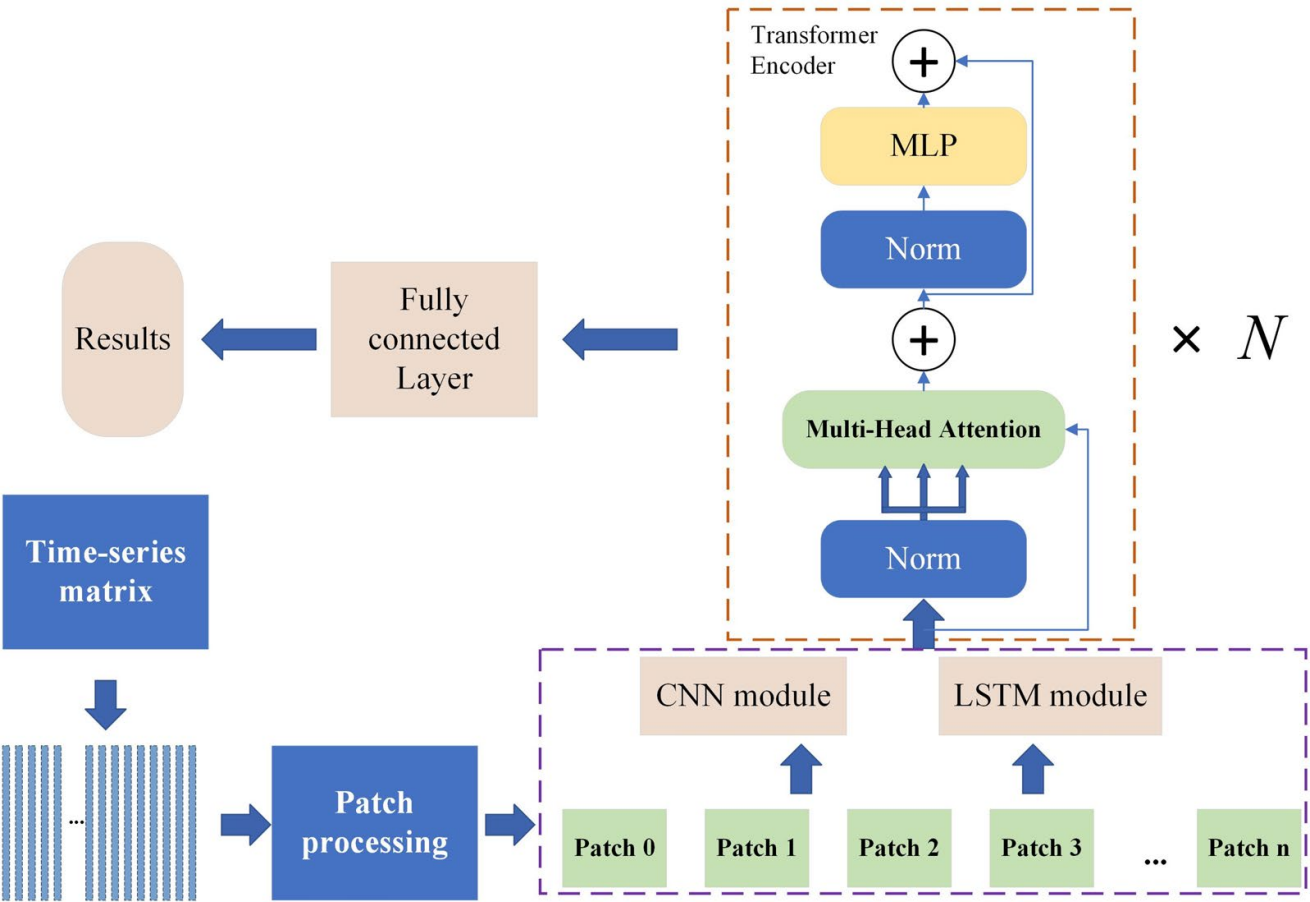
The architecture of CNN-Transformer and LSTM-Transformer

Traditional Transformer networks, when dealing with two-dimensional input matrices, typically embed each element of the 2D matrix into a high-dimensional space and then employed the Transformer's self-attention mechanism to capture dependencies between these elements. In the conventional architecture of the Transformer network, the Encoder layer typically commences with an Embedding layer. This Embedding layer is responsible for transforming each time-series feature into a vector representation, subsequently serving as the input for the Transformer. In this experiment, we transform time-series data matrices based on the model's input feature quantity N and a maximum of 300 time points, then replace the traditional Embedding layer with either an LSTM network or a CNN network to combine and extract features from the time-series data to enhance feature diversity and richness. Within the Transformer's Encoder module, the input data undergoes self-attention mechanisms to extract both local and global features from the time-series data. Ultimately, classification results are derived through the utilization of fully connected layers and a SoftMax layer. The detailed network architecture is outlined below (Fig. 3):

The main contribution of this study lies in introducing a novel time-series data modeling approach specifically designed for sepsis patients. This approach effectively accommodates time-series data of varying lengths and frequencies. Our goal is to facilitate early and precise sepsis prediction, consequently extending the crucial timeframe for early intervention and treatment of sepsis patients. Furthermore, this method upholds a robust predictive performance.

## Statistics

Categorical variables are presented as counts and percentages, and continuous variables are presented as mean and standard deviation (SD). Comparisons between groups were performed by 2-tailed t-test for continuous variables and chi-square test for categorical variables. All statistical analyses were performed in the python package SciPy (SciPy) [18]. The statistical significance was considered as *P* < 0.5.

## SHAP-Visualization

In 2017, Lundberg proposed the SHAP (Shapley additive explanations) method. SHAP exhibits additivity consistency in explaining the output results [19], consistent with the general notion of regression. For each predicted sample, the model generates a predicted value, and the SHAP value is the assigned numerical value to each feature in that sample. Assuming the *i-th* sample is denoted as *x*_*i*_, the *j-th* feature of the *i-th* sample is denoted as *x*_*ij*_, the model's predicted value for the *i-th* sample is *y*_*i*_, and the mean of all sample predictions is denoted as *y*_*base*_. Then, the SHAP value of x_ij_ follows the following equation:

1 $${y}_{i}={y}_{base}+f\left({x}_{i},1\right)+f\left({x}_{i},2\right)+\dots +f\left({x}_{i},k\right)$$

Here, $f\left({x}_{i},j\right)$ represents the SHAP value of *x*_*ij*_. This feature ensures that the sum of the contribution values equals the final output, thereby eliminating the interpretability differences caused by structural variations among different models.

The main idea behind SHAP is derived from the Shapley value in cooperative game theory. Shapley developed this method to address the problem of allocating cooperative benefits among multiple players. In a set *I* = {1,2,…,*n*} of *n* participants, if there exists a cooperative benefit function *v* for any subset *S* of *I* that satisfies *v*($\varnothing$) = 0 and for any disjoint subsets *S*_*1*_ and *S*_*2*_ of *I* exist *v*(*S*_*1*_ $\cup$ *S*_*2*_) $\ge$ *v*(*S*_*1*_) + *v* (*S*_*2*_), then for each participant involved, the allocation of their contribution *x* = {*x*_*1*_,*x*_*2*_,…,*x*_*n*_} must satisfy the following conditions for cooperation to occur: $\sum {x}_{i}=v\left(i\right) i=\mathrm{1,2},\dots ,n$, *x*_*i*_ ≥ *v*(*i*), *i* = 1,2,…,*n*. In other words, each participant should receive no less than their individual contribution in a non-cooperative scenario, and the sum of the allocations should equal the total benefit. For the benefit function *v*, the allocation $\varphi \left(v\right)=({\varphi }_{1}\left(v\right),{\varphi }_{2}\left(v\right),\dots ,{\varphi }_{n}(v))$ has been proven to satisfy the following properties:

2 $${\varphi }_{i}\left(v\right)= \sum\nolimits_{\begin{array}{c}T\in I\\ i\in T\end{array}}\frac{\left(\left|T\right|-1\right)!\left(n-\left|T\right|\right)!}{n!}\left(v\left(T\right)-v\left(T-\left\{i\right\}\right)\right)$$

Here, *T* is a subset of the set, |*T*| represents the number of elements in the subset, and *n* represents the total number of members. SHAP builds upon the Shapley value and makes improvements suitable for Machine learning models. It treats features as players and model outputs as the cooperative results. For the *K-th* data point, the model output can be represented as *v*_*k*_(*I*), The contribution value of each feature at that data point, denoted as ${\varphi }_{i}\left({v}_{k}\right)$, is also known as the SHAP value. Unlike linear models that use the magnitude of parameters or coefficients to measure the contribution of a feature to the model, the SHAP algorithm calculates the combined contributions of each feature for every sample. In the end, we obtain the contribution of each feature in each sample. If a feature exhibits consistent trends across most samples, it indicates that the model recognizes the importance of that feature in either a positive or negative direction.

## Results

### Patient characteristics

The cohort included 17,932 ICU patients, of whom 9092 patients (50.70%) developed sepsis. The mortality rate of sepsis patients is significantly higher compared to regular ICU patients, showing a notable difference (Sepsis: 14.19% vs. Non-Sepsis: 7.86%). Furthermore, the BMI index of sepsis patients is also significantly lower than that of regular ICU patients (Sepsis: 15.72 ± 5.29 vs. Non-Sepsis: 20.67 ± 6.62), highlighting the substantial harm that sepsis poses to patient prognosis. Regarding patient age, there was no statistical significance (*P* > 0.05). The basic demographic characteristics of the cohort are presented in Table 1.

**Table 1:** Base characteristics of the included patients

***Variables (mean* ± *SD)***	***Non-Sepsis***	***Sepsis***	***P***
***Sex***			0.011*
*** Female***	3980 (45.02%)	4286 (47.14%)	
*** Male***	4860 (54.97%)	4806 (52.86%)	
***Age(yrs)***	65.32 ± 16.05	65.29 ± 16.06	> 0.05
***BMI***	20.67 ± 6.62	15.72 ± 5.29	< 0.001
***Discharge status***			< 0.001*
*** Alive***	8144 (92.13%)	7800 (85.79%)	
*** Expired***	695 (7.86%)	1290 (14.19%)	
***Unit type***			< 0.001*
*** Cardiac ICU***	569 (6.44%)	802 (8.82%)	
*** CCU-CTIU***	403 (4.56%)	506 (5.57%)	
*** CSICU***	1226 (13.87%)	194 (2.13%)	
*** CTICU***	461 (5.21%)	57 (0.63%)	
*** Med-Surg ICU***	5233 (59.20%)	6044 (66.48%)	
*** MICU***	292 (3.30%)	918 (10.10%)	
*** Neuro ICU***	614 (6.95%)	208 (2.29%)	
*** SICU***	42 (0.48%)	363 (3.99%)	
***ICU stay***	3.27 (2.35)	5.76 (6.74)	< 0.001
***Apachescore***	64.27 (27.54)	73.87 (27.36)	< 0.001s

^*^ Chi-square test

### Model performance

**Fig. 4:**
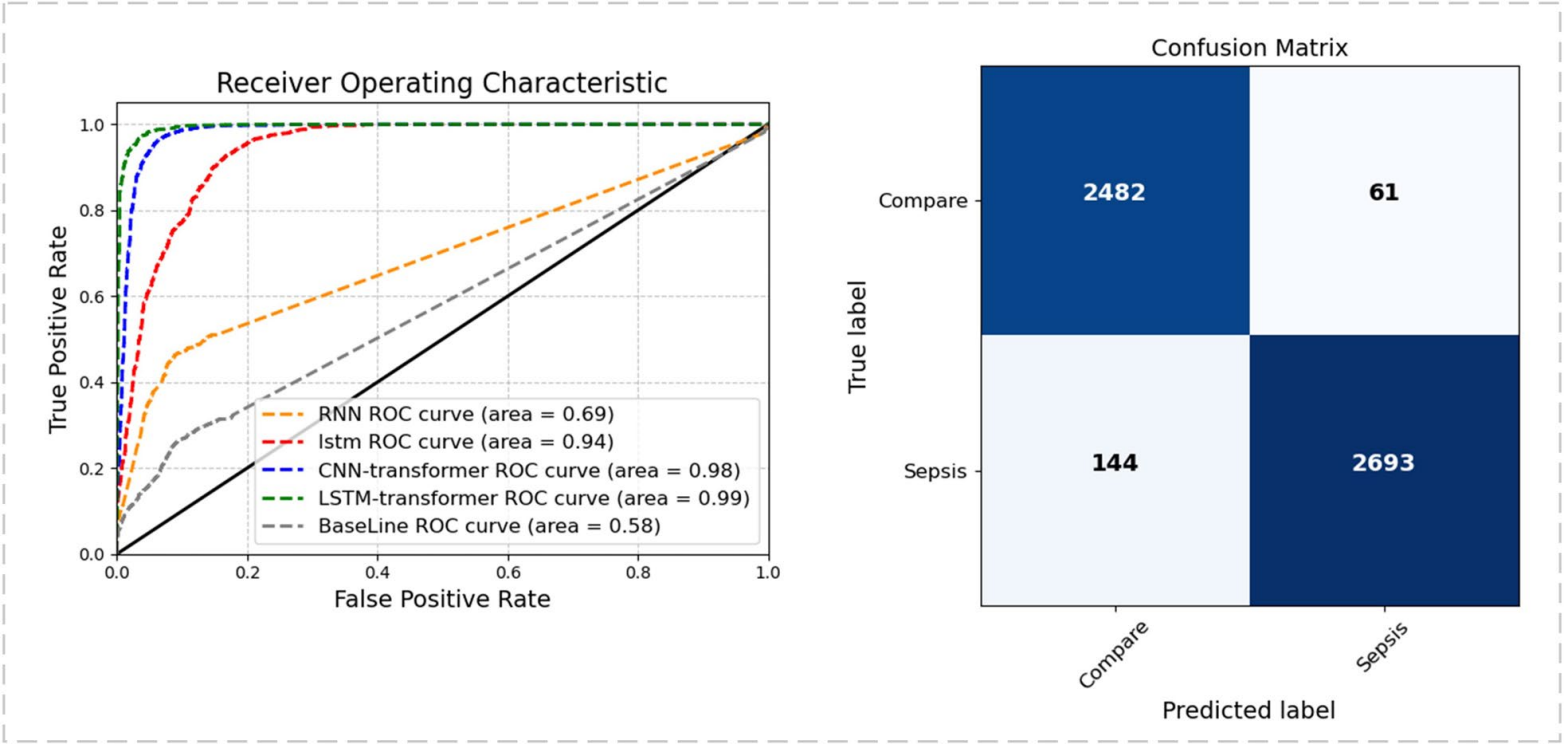
The results of different models and the confusion matrix of LSTM-transformer

With a 12-h time window selected, Fig. 4 displays the area under the receiver operating characteristic (AUROC) curves for these predictive models. Among the three models, the LSTM-Transformer exhibited the highest performance, reaching up to 0.99. Based on the LSTM-Transformer model, the specific model classification results are presented using a confusion matrix (as shown below). In this investigation, we introduce time windows and employ Transformer self-attention modules to holistically scrutinize their impact on model performance. We scrutinize the clinical metrics obtained during the patients' inaugural hospitalization, and concomitantly integrate them with conventional recurrent neural networks to formulate our foundational model. This culmination yields an AUROC value of 0.58, serving as a baseline for our research.

In addition, we employed various statistical metrics to evaluate and compare the results among different models on the same test dataset, including accuracy, precision, recall, F1 score, and others. Additionally, based on 5-fold cross-validation, the average results of the improved Transformer-based model indicate a significant enhancement in all performance metrics (Table 2).

**Table 2:** The outcomes of prediction models(mean ± std)

**Model**	**Baseline**	**Time window + RNN**	**Time window + LSTM**	**Time window + CNN + Transformer**	**Time window + LSTM + Transformer**
**Accuracy**	0.637 (0.035)	0.653 (0.032)	0.835 (0.023)	0.951 (0.017)	0.964 (0.018)
**Precision**	0.711 (0.037)	0.715 (0.030)	0.875 (0.047)	0.955 (0.028)	0.956 (0.012)
**Recall**	0.677 (0.044)	0.691 (0.041)	0.861 (0.011)	0.951 (0.012)	0.967 (0.012)
**F1-score**	0.667 (0.045)	0.662 (0.039)	0.871 (0.054)	0.954 (0.023)	0.959 (0.014)

**Fig. 5:**
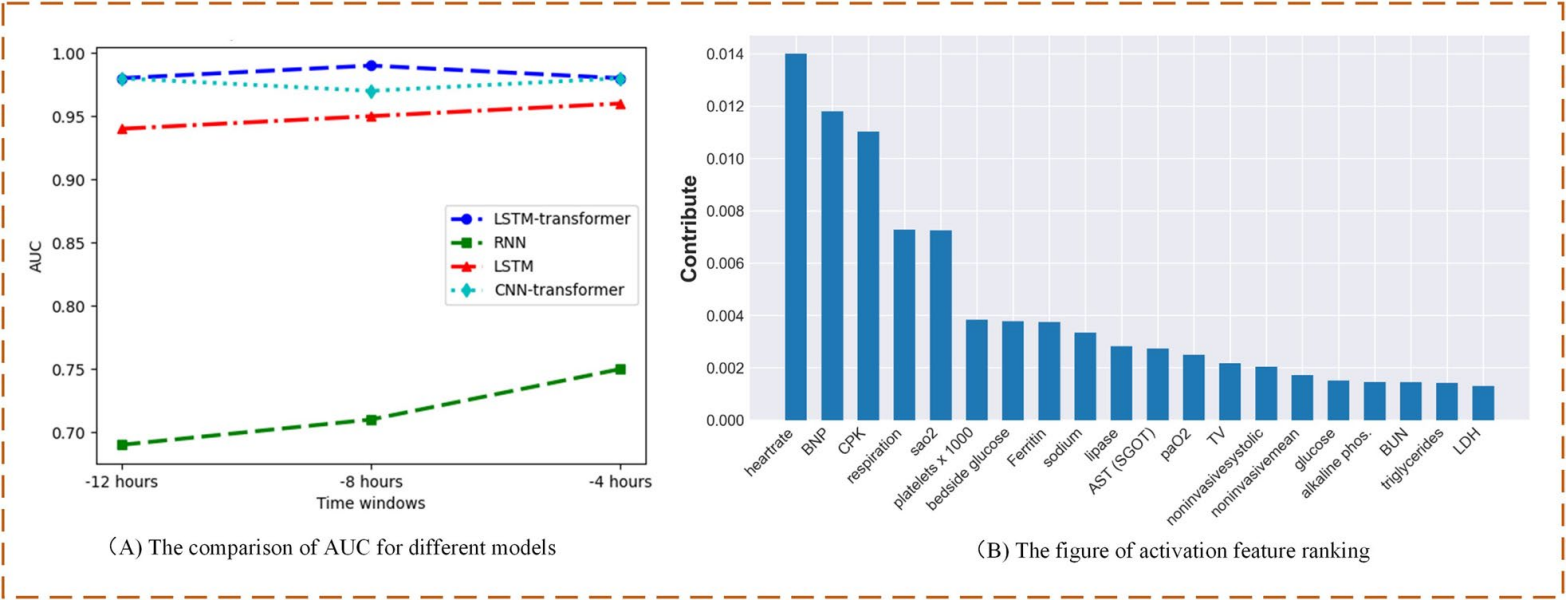
**A** Results of different models at different time windows. **B** The activation feature ranking based on the LSTM-transformer model in the 12-h window

By adjusting different time windows and collecting time-series data from patients in the 4-h and 8-h periods before sepsis diagnosis, we compared the predictive performance of different network models. It can be observed that our LSTM-Transformer model exhibited favorable performance as early as the 12-h window, and as we extended the review window, the improvement in model performance was not particularly significant (Fig. 5A). Based on the prediction model, weight analysis is conducted using the SHAP algorithm, which generates visual heatmaps. The Fig. 5B displays the activation feature ranking based on the LSTM-transformer model in the 12-h window.

**Fig. 6:**
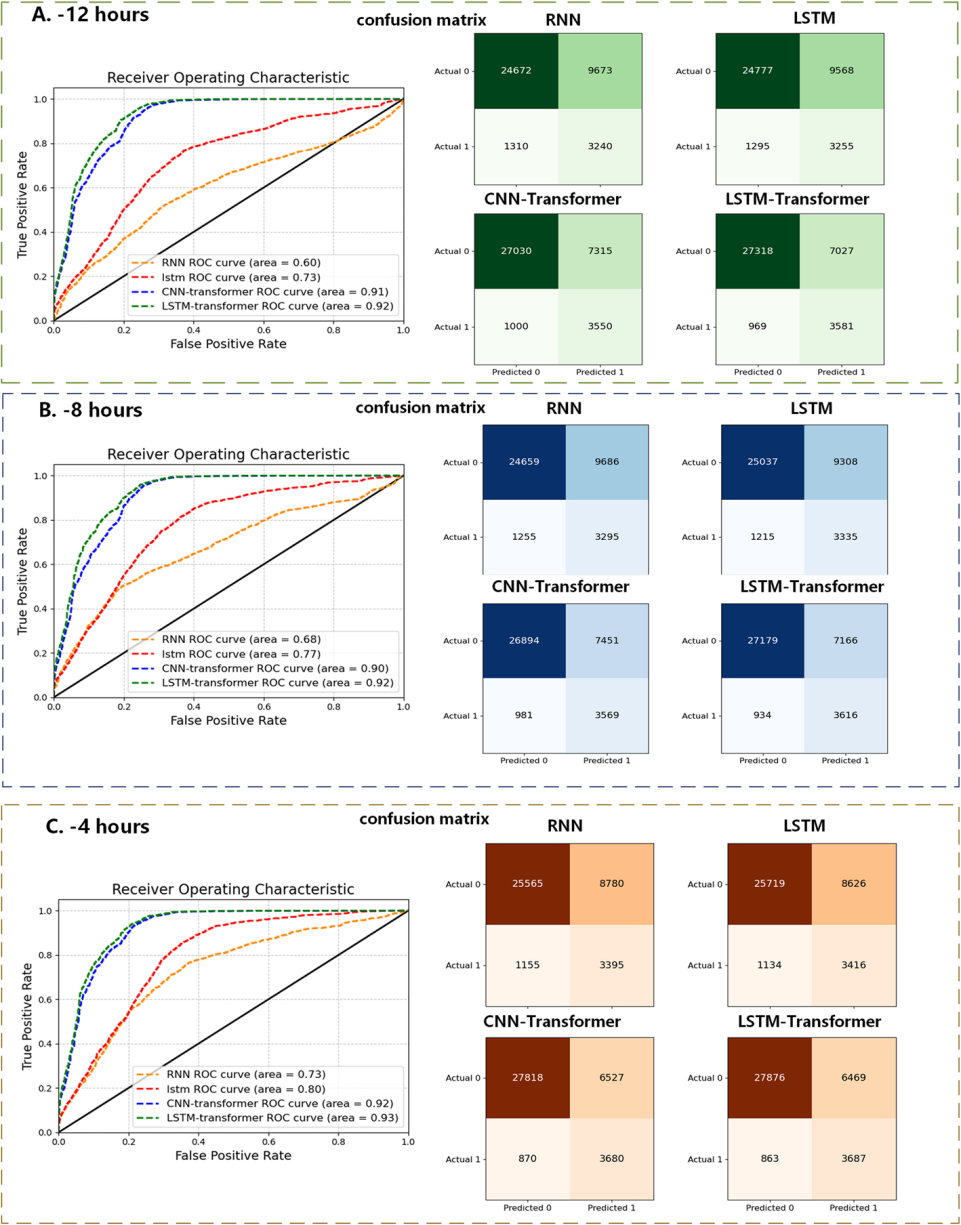
The external validation results on the MIMIC dataset (0: Non-sepsis, 1: Sepsis)

Considering the generalization of the model, we utilized data from MIMIC (Medical Information Mart for Intensive Care) for external validation. The overall accuracy has experienced a modest decrease, with an accuracy of approximately 80% and the AUC value still hovers around 0.9 in the time window (advance 4 fours) + LSTM-Transformer model. The predicted results for different time windows are as shown in Fig. 6.

## Hospital system integration with predictive models

**Fig. 7:**
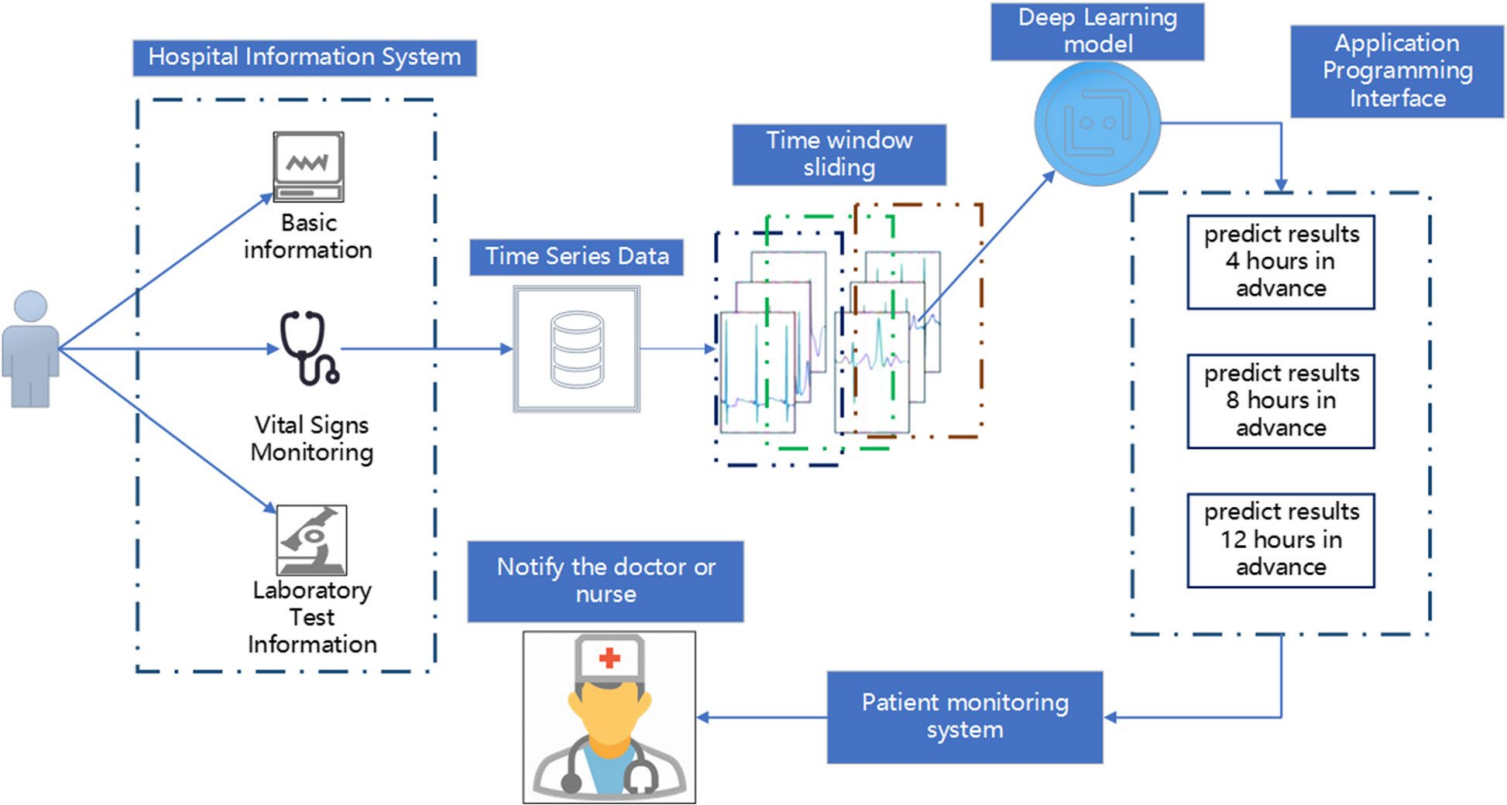
The framework of hospital system integration with predictive models

In our research, we are also exploring integration with hospital information systems by collecting real-time patient data for model testing, with the goal of providing meaningful assistance to clinical sepsis patients. To achieve this, we have devised the following system integration framework, illustrated in Fig. 7: gathering patient's fundamental information, laboratory test results, and vital signs monitoring values since admission, and consolidating this data in a centralized data center. Considering that patients generate time-series data every minute, it is impractical to continually assess their sepsis risk. Therefore, we propose setting an interval, such as five or ten minutes, to collect patient data using a 300 * N time window. This data is then processed through an Application Programming Interface (API) to invoke deep learning models, providing predicted outcomes for 4 h, 8 h, and 12 h back to the patient monitoring system. The final decision on whether to clinically intervene rests with the doctor, thus completing the entire closed-loop process. Currently, we have finalized the framework design, however, the implementation of clinical applications entails tasks such as data integration, system integration, and obtaining ethical approvals. This process necessitates some additional time.

## Discussion

Machine learning is considered a promising approach for sepsis prediction in the ICU. The key to sepsis prevention is the early identification and treatment of the underlying causes of the inflammatory response. Early diagnosis and timely intervention in sepsis patients can significantly improve outcomes. Therefore, there is an urgent need for an accurate and efficient sepsis bedside early prediction tool. In this study, we constructed a model for early prediction of sepsis in ICU patients. By collecting time-series data from patients in the 4-h, 8-h, and 12-h periods before sepsis diagnosis and inputting it into different network models for comparative analysis, we found that the time-series model based on the Transformer architecture demonstrated outstanding predictive capabilities in an earlier time window (i.e., 12 h before onset). This finding provides an opportunity for earlier clinical intervention, which has the potential to result in significant enhancements in disease management and patient care. As the time window gradually shifts backward, traditional recurrent neural network models also progressively exhibit performance improvements. This implies that in the later stages of disease progression, traditional models gradually approach the advantages exhibited by Transformer-based models in the early stages. It highlights the potential of Transformer-based time-series models for early prediction, offering a more forward-looking tool for clinical practice. However, it's important to note that the gradual improvement of traditional recurrent neural networks in later-stage prediction may stem from their enhanced capability to capture long-term temporal relationships. Therefore, by considering the strengths of both models, it is possible to develop more precise and individualized treatment plans for healthcare teams, potentially leading to greater impacts on patient health outcomes.

A sufficiently large dataset is key to training machine learning models for achieving good performance. The inclusion of new tests and technologies can enhance our prediction tasks, but their direct integration into existing machine learning models is often impractical. Therefore, transfer learning may be a promising and viable strategy to maintain the effectiveness of machine learning models across multi-center deployments. Chen et al. improved the performance of LightGBM and MLP models in predicting sepsis occurrence within 1–5 h using Transformer, achieving favorable areas under the receiver operating characteristic curve (AUC) within the range of 0.96–0.98 [20]. In our study, the process of transfer learning effectively enhanced the performance of RNN and LSTM models in predicting sepsis occurrence in the e-ICU dataset. When predicting sepsis occurrence 12 h in advance, compared to traditional RNN and LSTM models, the CNN-Transformer and LSTM-Transformer models demonstrated a clear advantage. Among these, the LSTM-Transformer showed the best area under the curve (AUC) of 0.99, accuracy of 95.6%, and recall of 0.967. Furthermore, transfer learning has been applied to similar domains or tasks in multiple medical fields, reducing the requirements for target dataset size while enhancing training speed and prediction performance [21, 22]. The results of this study clearly demonstrate the potential benefits of Transformer models in clinical prediction and underscore the critical role of model selection within specific time windows. This exploratory research not only brings new perspectives to medical research but also provides valuable insights for the future development of healthcare practices. Sepsis often leads to organ dysfunction and damage, and acute respiratory distress syndrome (ARDS) can exacerbate acute injuries. Therefore, ARDS is typically considered a fatal consequence of severe sepsis, accounting for approximately 32% of all cases of sepsis [23]. ARDS usually manifests as a sudden exacerbation of non-cardiogenic pulmonary edema, severe hypoxemia, and requires mechanical ventilation to improve oxygenation [24]. For patients admitted to the ICU due to respiratory system diseases, especially COVID-19, respiratory parameters are closely related to ICU mortality. Plateau pressure, which is the pressure inside the alveoli during breath-holding positive pressure ventilation, has been analyzed for its impact on the prognosis of ARDS patients [25]. Timely monitoring of a patient's respiratory condition plays a positive role in improving their prognosis.

Enhancing the interpretability of data-driven models can help overcome the barriers to trust and acceptance of these machine learning models by practitioners in clinical settings. In this study, SHAP analysis served as an interpretive tool, aiding healthcare professionals in identifying key risk factors. In our study, the importance of variables showed that heartbeat rate, BNP, AST, respiration, Sao2, and palates were the most important risk factors that contribute to the predicted occurrence of sepsis. The concept of heart rate analysis has been around for decades, and with the continuous improvement in computing power, it has become increasingly important [26]. Atrial fibrillation (AF) is a common complication of sepsis, and continuous heart rate variability monitoring contributes to the rapid diagnosis and early intervention of severe sepsis, altering the course of sepsis-related conditions [27]. Additionally, heart rate is another important factor in predicting the occurrence of sepsis-related acute brain injury [28]. Furthermore, elevated BNP levels have practical applications in the early diagnosis, clinical treatment, and prognosis assessment of severe sepsis patients. BNP is one of the members of the natriuretic peptide family secreted by the heart. It promotes diuresis and sodium excretion, effectively dilates blood vessels, and anti-natriuretic peptide is an important marker for assessing heart damage [29]. The excessive inflammatory stress response in sepsis generates more cardiac toxins, and infections by pathogenic microorganisms can also produce more endotoxins, thereby inducing an increase in BNP levels in the body [30]. Sepsis can lead to multi-organ damage and even failure. During systemic infection, the cytokine storm and endotoxin production in sepsis can damage liver cells, resulting in organ dysfunction. Damaged liver cells release damage-associated molecular patterns, triggering a more severe systemic inflammatory response, and in severe cases, it can lead to death [31]. Abnormal levels of serum aspartate aminotransferase (AST) are sensitive indicators of liver cell damage [32]. Early testing of liver function parameters before the onset of sepsis can improve patient survival to some extent. Furthermore, sepsis is associated with an increased platelet reactivity [33]. There is evidence to suggest that cytokines released during sepsis can directly activate platelets [34]. Sepsis is also related to an increase in bone marrow platelet release, leading to thrombocytosis, which is mediated by elevated levels of platelet growth factors and cytokines such as interleukin-6 [35].

## Limitations

The limitations of this experiment include the fact that both model training and validation were based on the same publicly available dataset. When externally validating with the MIMIC dataset, there was a certain degree of decline observed in its predictive performance. In the future, we will consider incorporating data from additional sources while integrating this model with hospital information systems to validate its effectiveness. Additionally, in terms of model interpretability, we have only validated the overall associations between clinical indicators and sepsis occurrence within the predefined 12-h window. Visual analysis at specific time points and the interpretation of individual patient clinical information have not been implemented yet.

## Data Availability

The data used in this study were sourced from the publicly available eICU database (https://eicu-crd.mit.edu/about/eicu/). For access to the related code, please contact the corresponding author.
